# Association between TNF α Gene Polymorphisms and the Risk of Duodenal Ulcer: A Meta-Analysis

**DOI:** 10.1371/journal.pone.0057167

**Published:** 2013-02-22

**Authors:** Bei-Bei Zhang, Xing-Zhen Liu, Jin Sun, Yan-Wei Yin, Qian-Qian Sun

**Affiliations:** 1 Department of Medical Affairs, General Hospital of PLA Chengdu Military Area Command, Chengdu, China; 2 Department of Endocrinology, Changhai Hospital, Second Military Medical University, Shanghai, China; 3 Department of Gynecological and Obstetrical, General Hospital of PLA Chengdu Military Area Command, Chengdu, China; 4 Department of Emergency, the Chinese PLA Air Force General Hospital, Beijing, China; 5 Jinsong Sanatorium of Beijing Air Force, Beijing, China; University of California, Riverside, United States of America

## Abstract

**Background:**

Epidemiological studies have evaluated the association between tumor necrosis factor α (TNF-α) single nucleotide polymorphisms (SNPs) and duodenal ulcer (DU), but the results remain inconclusive. The aim of this study was to perform a meta-analysis to investigate a more authentic association between TNF-α SNPs and DU.

**Methods:**

We performed the meta-analysis by searching PubMed, Embase, and Web of Science databases from the first available year to Sep. 5, 2012. Additionally, checking reference lists from identified articles, reviews, and the abstracts presented at related scientific societies meetings were also performed. All case-control studies investigating the association between TNF-α SNPs and DU risk were included. The association was assessed by odds ratio (OR) with 95% confidence interval (CI). Publication bias was analyzed by Begg's funnel plot and Egger's regression test.

**Results:**

A total of sixteen studies reporting TNF-α −308G/A, −1031T/C, −863C/A, −857C/T, and −238G/A polymorphism were included in our final meta-analysis. There was no statistically significant association between −308G/A polymorphism and DU in the overall study population, as well as subgroup analyses by ethnicity, study design, and H. pylori status. As for −1031T/C, −863C/A, −857C/T, and −238G/A, results of our meta-analyses showed no statistical evidence of significant association. Power calculation on the combined sample size showed that the statistical powers were all lower than 80% for all the meta-analyses.

**Conclusions:**

The data suggests that there is no statistical evidence of significant association between the studied TNF-α SNPs and DU. However, this conclusion should be interpreted with caution as low statistical powers were revealed by power calculations. In future, larger sample-size studies with homogeneous DU patients and well-matched controls are required.

## Introduction

Duodenal ulcer (DU) is one type of peptic ulcer that occurs in the duodenum. DU may result from infection of Helicobacter pylori (H. pylori) bacteria, overuse of alcohol, and medications such as aspirin and non-steroidal anti-inflammatory drugs (NSAIDs) [1], [2]. Both H. pylori infection and NSAIDs can increase gastric acid secretion in the stomach and also degrade the mucus barrier, and finally allow gastric acid to make contact with the duodenal lining, leading to inflammation and eventually ulceration. However, even as the most common cause of DU, the H. pylori infection only accounts for less than 20% of infected individuals who develop DU [3]. Possible explanation for this phenomenon is that the nature of the host immune response influences the clinical outcome [4]. Taking all these into consideration, we naturally come to the conclusion that DU is a multi-factorial disease involving environmental factors and host genetic factors.

With respect to host genetic factors, our previous studies demonstrated no association between DU and interleukin (IL)-1 β 511 C/T and 31 C/T polymorphisms, but significant association between IL-8 −251 T/A polymorphism and DU [5]–[7]. In addition to the above factors, tumor necrosis factor α (TNF-α) has been reported to have the proinflammatory activity and the capability of inhibiting gastric acid secretion [8]–[10]. TNF-α, which is produced by macrophages, monocytes, neutrophils, T-cells, and NK-cells after stimulation, is a pro-inflammatory cytokine and plays a role in cell immunity. The TNF-α gene is located in the class III region of the major histocompatibility complex on chromosome 6. It is suggested that polymorphisms in the regulatory region can influence the expression of TNF-α [11], and thereby increase the susceptibility of H. pylori infection. In 1999, Kunstmann et al. firstly reported the association of specific genotype of the TNF-α gene with the susceptibility to DU [12]. Thereafter, a variety of epidemiological studies have evaluated the association between DU and TNF-α gene polymorphisms, including −308G/A, −1031T/C, −863C/A, −857C/T, −238G/A, −376 G/A, and −806 C/T [13]–[30]. However, results of different studies have been inconsistent. In addition, the sample size in each of published studies was relatively small, which limited the credibility of results. The present meta-analysis was designed to derive a more precise estimation of the association between TNF-α single nucleotide polymorphisms (SNPs) and DU.

## Materials and Methods

### Literature search

This meta-analysis followed the Meta-analysis of Observational Studies in Epidemiology (MOOSE) guidelines [31] and Preferred Reporting Items for Systematic Reviews and Meta-analyses (PRISMA) criteria [32]. We collected all published studies on humans up to Sep. 5, 2012 by systematically searching the PubMed, Embase, and ISI Web of Science with the search terms: (“tumor necrosis factor α” OR “TNF-α”) AND (“polymorphism” OR “mutation” OR “variant” OR “genotype”) AND (“duodenal ulcer” OR “peptic ulcer”). The language was limited to English. The electronic searching was supplemented by checking reference lists from identified articles, reviews and the abstracts presented at related scientific societies meetings. Two investigators (Zhang BB and Yin YW) screened each of the titles, abstracts, and full texts to determine inclusion independently. The results were compared and disagreements were resolved by consensus.

### Inclusion criteria

The inclusion criteria were as follows: (1) studies on the relationship between TNF-α gene polymorphisms and DU, including −308G/A, −1031T/C, −863C/A, −857C/T, −238G/A, −376 G/A, and −806 C/T; (2) published case-control studies; (3) studies with full text articles; (4) sufficient data for estimating an odds ratio (OR) with 95% confidence interval (CI).

### Data extraction

The information was carefully extracted by the same two authors (Zhang BB and Yin YW) according to the inclusion criteria listed above independently. Disagreement was resolved by consensus. If these two authors could not reach a consensus, another author (Sun QQ) was consulted. The following data were collected from each study: first author's name, publication date, country, ethnicity, study design (source of controls), and evidence of Hardy-Weinberg equilibrium (HWE) (*P*<0.05 of HWE was considered significant), respectively. Different ethnicities were categorized as Caucasian, Asian, African, American Indian, and mixed. Study design was stratified to population-based (PB) studies and hospital-based (HB) studies. Total numbers of cases and controls, and frequency of −308G/A, −1031T/C, −863C/A, −857C/T, −238G/A, −376 G/A, and −806 C/T mutation in cases and controls regardless of H. pylori status were extracted. When studies reported genotype distributions for H. pylori negative and H. pylori positive only, we also extracted data of each group separately for subgroup analyses.

### Quality score assessment

Two authors (Zhang BB and Yin YW) of this article independently assessed the qualities of included studies using the Newcastle-Ottawa Scale (NOS) [33]. The NOS ranges between zero (worst) up to nine points (best). We assessed included studies based on three aspects: the selection of the study groups (maximum of 4 points: whether the case definition is adequate with independent validation; whether the patients are consecutive or obviously representative series of cases; whether the controls are population-based; whether the controls are healthy person without history of duodenal ulcer); the comparability of the groups (maximum of 2 points: whether cases and controls are matched for gender and age; whether cases and controls are matched for region or ethnicity); and the ascertainment of the exposure (maximum of 3 points: whether the ascertainment of exposure is secure record; whether the same method of ascertainment is applied for cases and controls; whether the same non-response rate is reported for both groups). Studies with a score of seven points or greater were considered to be of high quality. Disagreement was settled as described above.

### Statistical analysis

Combined ORs with their 95% CIs were calculated respectively for four genetic models: allelic model (2 allele vs. 1 allele), additive model (2/2 vs. 1/1), dominant model (1/2+2/2 vs. 1/1), and recessive model (2/2 vs. 1/2+1/1), in which 2 indicates the minor allele [34]. Between-study heterogeneity was assessed by the Q-test and I^2^ statistic, *P*<0.10 and I^2^>50% indicated evidence of heterogeneity [35], [36]. The ORs were pooled through a fixed effects model, using the Mantel-Haenszel approach when no heterogeneity was observed among studies [37]. Otherwise, a random effects model was adopted [38]. Publication bias was analyzed by Begg's funnel plot and Egger's test (*P*<0.05 was considered representative of statistically significant publication bias) [39]. All above statistical analyses were performed using Stata 11.0. Power analysis was performed using Quanto software package (Version 1.2.4, http://hydra.usc.edu/gxe/) [40]. Unmatched case-control design, gene only hypothesis and additive inheritance model were used. We assumed a population risk of 0.12 for DU and considered a two-sided p-value of 0.05.

## Results

### Study characteristics

**Figure 1:**
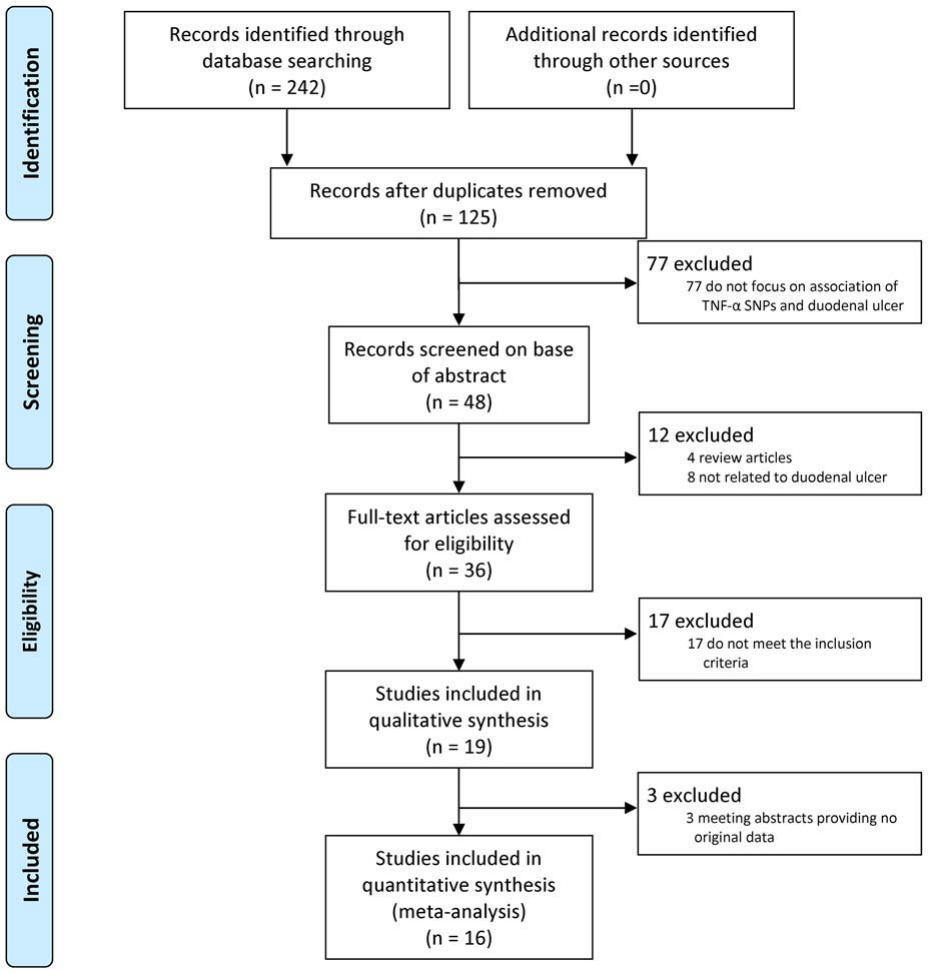
Flow diagram of the study selection process.

The present study met the PRISMA statement requirements (**Table S1** and **Fig. 1**). Two hundred and forty-two potentially relevant studies were identified after the searching. Based on our inclusion criteria, a total of nineteen studies were included in qualitative synthesis [12]–[30]. Three studies were excluded from further meta-analysis as they provided no origin data for calculating ORs and 95%CIs [15], [18], [28]. Hence, sixteen articles were included in the meta-analysis [12]–[14], [16], [17], [19]–[27], [29], [30]. **Table 1** shows the main characteristics of the studies included in meta-analysis. Sixteen studies evaluated the relationship between −308G/A polymorphism and DU. Five studies reported association between DU and −1031T/C, −857C/T, and −238G/A polymorphism. There were four studies related −863C/A. Only one study reported the relationship between −376 G/A, −806 C/T polymorphism and DU, so both SNPs were not meta-analyzed. Considering sufficient data for −308G/A, we performed subgroup analyses by ethnicity (Asian and Caucasian), source of controls (population-based and hospital-based), and H. pylori status (H. pylori negative and H. pylori positive).

**Table 1:** Main characteristics of studies included in the meta-analysis.

First author	Year	Country	Ethnicity	SOC	SNP	Genotypes distribution			MAF	HWE Y/N(*P*)	Score
						case/control, N(%)			case/control, N(%)		
						1/1	1/2	2/2	2		
Kunstmann	1999	Germany	Caucasian	HB	−308G/A	14(100.0)/68(69.4)	0(0.0)/22(22.4)	0(0.0)/8(8.2)	0(0.0)/38(19.4)	N(0.005)	7
Lanas	2001	Spain	Caucasian	PB	−308G/A	93(72.1)/83(82.2)	34(26.4)/16(15.8)	2(1.5)/2(2.0)	38(14.7)/20(9.9)	Y(0.26)	9
					−238G/A	108(83.7)/85(84.1)	21(16.3)/14(13.9)	0(0.0)/2(2.0)	21(8.1)/18(8.9)	Y(0.14)	
Gyulai	2004	Hungary	Caucasian	PB	−308G/A	54(78.3)/29(61.7)	13(18.8)/16(34.0)	2(2.9)/2(4.3)	17(12.3)/20(21.3)	Y(0.91)	9
Lee	2004	Korea	Asian	PB	−308G/A	115(86.5)/218(83.5)	18(13.5)/42(16.1)	0(0.0)/1(0.4)	18(6.8)/44(8.4)	Y(0.49)	8
					−238G/A	118(88.7)/236(90.4)	15(11.3)/25(9.6)	0(0.0)/0(0.0)	15(5.6)/25(4.8)	Y(0.42)	
					−857C/T	105(78.9)/185(70.9)	23(17.3)/69(26.4)	5(3.8)/7(2.7)	33(12.4)/83(15.9)	Y(0.85)	
					−863C/A	88(66.2)/181(69.3)	37(27.8)/72(27.6)	8(6.0)/8(3.1)	53(19.9)/88(16.9)	Y(0.80)	
					−1031T/C	76(57.1)/161(61.7)	46(34.6)/92(35.2)	11(8.3)/8(3.1)	68(25.6)/108(20.7)	Y(0.23)	
Garcia-Gonzalez	2005	Spain	Caucasian	PB	−308G/A	83(72.8)/81(83.5)	29(25.4)/15(15.5)	2(1.8)/1(1.0)	33(14.5)/17(8.8)	Y(0.75)	9
					−238G/A	96(84.2)/80(82.5)	18(15.8)/15(15.5)	0(0.0)/2(2.1)	18(7.9)/19(9.8)	Y(0.22)	
Lee	2005	Korea	Asian	PB	−308G/A	45(86.5)/103(85.8)	7(13.5)/17(14.2)	0(0.0)/0(0.0)	7(6.7)/17(7.1)	Y(0.40)	8
Li	2005	China	Asian	PB	−308G/A	66(84.6)/228(86.4)	11(14.1)/34(12.9)	1(1.3)/2(0.7)	13(8.3)/38(7.2)	Y(0.56)	9
Lu	2005	China	Asian	HB	−308G/A	71(82.6)/220(84.6)	15(17.4)/40(15.4)		-	NE	5
					−857C/T	65(72.2)/188(72.0)	25(27.8)/73(28.0)		-	NE	
					−863C/A	51(56.7)/216(82.1)	39(43.3)/47(17.9)		-	NE	
					−1031T/C	54(60.0)/219(83.9)	36(40.0)/42(16.1)		-	NE	
					−806C/T	84(98.8)/253(97.3)	1(1.2)/7(2.7)		-	NE	
Zambon	2005	Italy	Caucasian	HB	−308G/A	94(81.0)/496(77.0)	20(17.3)/138(21.4)	2(1.7)/10(1.6)	24(10.3)/158(12.3)	Y(0.91)	7
					−238G/A	103(88.8)/569(88.4)	13(11.2)/74(11.5)	0(0.0)/1(0.1)	13(5.6)/76(5.9)	Y(0.38)	
					−857C/T	67(57.8)/404(62.8)	38(32.8)/227(35.2)	11(9.4)/13(2.0)	60(25.9)/253(19.6)	N(0.003)	
					−1031T/C	67(57.8)/381(59.2)	45(38.8)/227(35.2)	4(3.4)/36(5.6)	53(22.8)/299(23.2)	Y(0.77)	
					−376G/A	109(94.0)/605(94.0)	7(6.0)/39(6.0)	0(0.0)/0(0.0)	7(3.0)/39(3.0)	Y(0.43)	
Kim	2006	Korea	Asian	PB	−308G/A	189(80.8)/400(86.8)	41(17.5)/59(12.8)	4(1.7)/2(0.4)	49(10.5)/63(6.8)	Y(0.91)	8
Sugimoto	2007	Japan	Asian	PB	−308G/A	92(97.9)/169(98.3)	2(2.1)/3(1.7)	0(0.0)/0(0.0)	2(1.1)/3(0.9)	Y(0.91)	8
					−857C/T	64(68.1)/125(72.7)	26(27.7)/40(23.3)	4(4.3)/7(4.0)	34(18.1)/54(15.7)	Y(0.11)	
					−863C/A	68(72.3)/125(72.7)	24(25.6)/44(25.6)	2(2.1)/3(1.7)	28(14.9)/50(14.5)	Y(0.70)	
					−1031T/C	68(72.4)/124(72.1)	24(25.5)/46(26.7)	2(2.1)/2(1.2)	28(14.9)/50(14.5)	Y(0.32)	
Wilschanski	2007	Israel	Caucasian	HB	−308G/A	-	-	-	6(26.1)/18(20.0)	NE	7
					−238G/A	-	-	-	10(43.5)/9(10.0)	NE	
Chakravorty	2008	India	Asian	PB	−308G/A	125(76.7)/107(72.8)	34(20.9)/35(23.8)	4(2.4)/5(3.4)	42(12.9)/45(15.3)	Y(0.32)	9
					−857C/T	152(93.3)/130(88.4)	11(6.7)/15(10.2)	0(0.0)/2(1.4)	11(3.4)/19(6.5)	Y(0.06)	
					−863C/A	108(66.3)/87(59.2)	47(28.8)/51(34.7)	8(4.9)/9(6.1)	63(19.3)/69(23.5)	Y(0.68)	
					−1031T/C	89(55.3)/60(40.8)	50(31.1)/65(44.2)	22(13.7)/22(15.0)	94(29.2)/109(37.1)	Y(0.53)	
Mei	2009	China	Asian	PB	−308G/A	365(83.5)/132(89.2)	68(15.6)/16(10.8)	4(0.9)/0(0.0)	76(8.7)/16(5.4)	Y(0.49)	9
Melo Barbosa	2009	Brazil	Mixed	HB	−308G/A	23(69.7)/86(86.0)	8(24.2)/13(13.0)	2(6.1)/1(1.0)	12(18.2)/15(7.5)	Y(0.53)	8
Cheng	2010	China	Asian	HB	−308G/A	114(85.7)/142(80.7)	17(12.8)/31(17.6)	2(1.5)/3(1.7)	21(7.9)/37(10.5)	Y(0.40)	6

HWE: Hardy-Weinberg equilibrium, Y: yes, N: no, NE: not evaluate, *P*: P value of test for HWE.

MAF: minor allele frequency.

SOC: source of control.

PB: population-based, HB: hospital-based.

SNP: single nucleotide polymorphism.

### Meta-analysis

**Figure 2:**
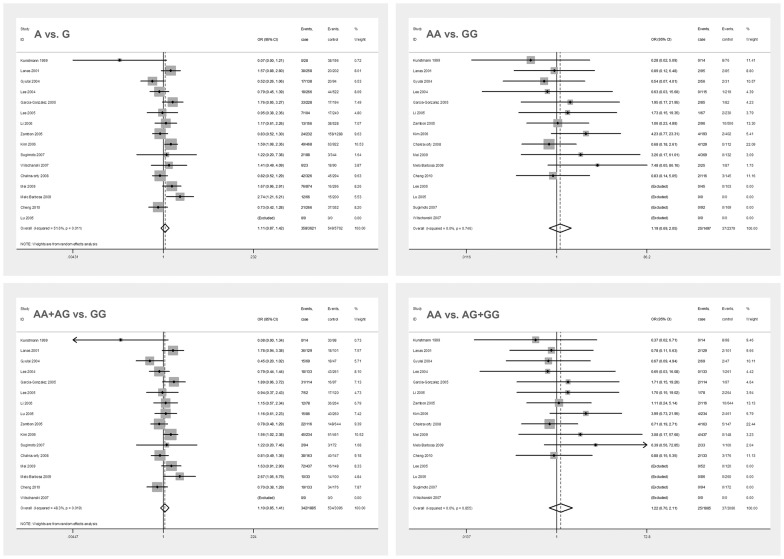
Forest plots of all models in overall studies for −308G/A. There was no statistical evidence of significant association between −308G/A and DU susceptibility. The summary pooled ORs and 95% CIs are indicated by the white diamond.

There were sixteen studies included in the analysis of −308G/A. The combined effects were as follows: for A allele vs. G allele: OR = 1.11, 95%CI = 0.87∼1.42; for AA vs. GG: OR = 1.19, 95%CI = 0.69∼2.05; for AA+AG vs. GG: OR = 1.10, 95%CI = 0.85∼1.41; and for AA vs. AG+GG: OR = 1.22, 95%CI = 0.70∼2.11 (**Fig. 2**). When meta-analysis was performed to assess association between −308G/A polymorphism and DU based on H. pylori status, no statistical evidence of significant association was found in all genetic models for both H. pylori positive subgroup and H. pylori negative subgroup. When stratified by ethnicity and study design, no statistically significant associations were found (**Table 2**). As for the association of TNF-α −238G/A, −1031T/C, −863C/A, −857C/T and DU susceptibility, there were no statistical evidence of significant association between all the above SNPs and DU susceptibility (**Fig. S1, S2, S3, S4**). Power calculation on the pooled frequencies showed that the statistical powers were all lower than 80% for all the above meta-analyses (**Table 2**).

**Table 2:** Results of meta-analysis for TNF-α SNPs and DU.

Study group	Study (N)	Sample size (case/control)	Allelic model		Additive model		Dominant model		Recessive model		Power calculation
			OR(95%CI)	M	OR(95%CI)	M	OR(95%CI)	M	OR(95%CI)	M	
−308G/A											
Overall	16	1885/3096	1.11(0.87∼1.42)	R	1.19(0.69∼2.05)	F	1.10(0.85∼1.41)	R	1.22(0.70∼2.11)	F	32.6%
PB	10	1503/1818	1.16(0.89∼1.50)	R	1.27(0.64∼2.52)	F	1.16(0.88∼1.54)	R	1.26(0.64∼2.50)	F	37.9%
HB	6	382/1278	1.03(0.55∼1.91)	R	1.05(0.42∼2.63)	F	0.97(0.56∼1.66)	R	1.14(0.45∼2.88)	F	5.7%
Caucasian	6	442/987	1.01(0.62∼1.67)	R	0.81(0.33∼1.95)	F	0.94(0.49∼1.82)	R	0.86(0.35∼2.09)	F	5.1%
Asian	9	1410/2009	1.10(0.90∼1.34)	F	1.34(0.63∼2.83)	F	1.09(0.87∼1.36)	F	1.34(0.63∼2.85)	F	18.0%
HP^−^	3	530/784	1.01(0.61∼1.67)	R	1.22(0.51∼2.93)	F	0.99(0.59∼1.66)	R	1.24(0.52∼2.98)	F	5.1%
HP^+^	8	865/1406	0.94(0.62∼1.43)	R	0.99(0.49∼2.02)	F	0.96(0.66∼1.41)	R	1.06(0.52∼2.16)	F	9.4%
−238G/A	5	492/1103	1.26(0.72∼2.23)	R	0.29(0.05∼1.68)	F	1.01(0.72∼1.42)	F	0.29(0.05∼1.66)	F	33.8%
−857C/T	5	596/1485	0.96(0.63∼1.46)	R	1.58(0.51∼4.94)	R	0.96(0.76∼1.20)	F	1.62(0.53∼5.00)	R	7.3%
−863C/A	4	480/843	0.99(0.78∼1.26)	F	1.19(0.62∼2.30)	F	1.32(0.69∼2.52)	R	1.23(0.64∼2.37)	F	5.1%
−1031T/C	5	594/1485	0.98(0.74∼1.29)	R	1.13(0.50∼2.54)	R	1.18(0.69∼2.03)	R	1.11(0.71∼1.73)	F	5.7%

M: model, F: fixed effects model, R: random effects model.

PB: population-based, HB: hospital-based.

H^+^: H. pylori positive, H^-^: H. pylori negative.

SNP: single nucleotide polymorphism.

DU: duodenal ulcer.

### Publication bias

The shapes of the funnel plots did not reveal any evidence of obvious asymmetry visually (**Fig. S5, S6, S7, S8, S9**). However, statistical evidence of publication bias were found using Egger's regression test for allelic model of −238G/A mutation and recessive model of −857C/T mutation. The results were as follows: for −308G/A mutation (*P* = 0.83 for allelic model, *P* = 0.22 for additive model, *P* = 0.98 for dominant model, and *P* = 0.23 for recessive model, respectively); for −238G/A mutation (*P* = 0.02 for allelic model, *P* = 0.89 for dominant model, respectively); for −1031T/C mutation (*P* = 0.84 for allelic model, *P* = 0.61 for additive model, *P* = 0.52 for dominant model, and *P* = 0.72 for recessive model, respectively); for −863C/A mutation (*P* = 0.98 for allelic model, *P* = 0.97 for additive model, *P* = 0.62 for dominant model, and *P* = 0.99 for recessive model, respectively); for −857C/T mutation (*P* = 0.18 for allelic model, *P* = 0.07 for additive model, *P* = 0.29 for dominant model, and *P* = 0.008 for recessive model, respectively).

## Discussion

TNF-α plays a key role in regulating gastric acid secretion, which is one of the most important factors in the development of duodenal diseases associated with H. pylori infection [9], [41], [42]. To date, many studies have evaluated the association between TNF-α SNPs and DU risk, but the results remain inconsistent. Also, the credibility of results from a single case-control study is questionable due to too small sample size of the study populations. Meta-analysis has the benefit to overcome this limitation by increasing the sample size and may generate more precise results, which has been widely used in genetic association studies [43], [44]. We therefore performed meta-analysis to assess whether combined evidence shows the association between TNF-α SNPs and DU.

Based on our study selection process, a total of nineteen publications were preliminarily eligible. Seven SNPs of TNF-α gene were reported, including −308G/A, −1031T/C, −863C/A, −857C/T, −238G/A, −376 G/A, and −806 C/T. Among these SNPs, only one study evaluated the association between −376 G/A and DU, which showed no significant association [30]; only one study reported the association between −806 C/T and DU, which demonstrated no significant association. Due to limited data, we did not carry out meta-analyses of these two SNPs.

In this meta-analysis, we identified eighteen articles focusing on the −308G/A polymorphism and its relationship with the risk of DU. Finally sixteen studies including 1,885 cases and 3,096 controls were included in the meta-analysis. The results of our meta-analyses demonstrated that there was no statistical evidence of significant association between the −308G/A polymorphism and DU in the overall study population regardless of the presence of H. pylori infection. These findings were consistent with most of the included studies as summarized in our meta-analyses. To explore a more precise relationship between −308G/A polymorphism and DU, we performed subgroup analyses by ethnicity, study design, and H. pylori status. First, we detected whether there was ethnic differences in the association between −308G/A polymorphism and DU. Our results demonstrated no statistically significant association among both Asian subgroup and Caucasian subgroup. When subgroup analyses were performed by study design, there was also no association. Considering H. pylori infection is the main cause of DU, we also performed meta-analysis to explore whether relationship between −308G/A polymorphism and DU would be associated with the status of H. pylori infection. However, no statistical evidence of significant association was shown in all models for both H. pylori-positive and H. pylori-negative subgroup.

As for −1031T/C, −863C/A, −857C/T, and −238G/A, four to five studies reported the association between them and the risk of DU, therefore meta-analyses were also performed. Among the studies included, one study demonstrated that −238G/A polymorphism might be a risk factor for DU in children infected with H. pylori [29]; one study showed that −857 TT genotype increased the risk of DU [30]; one study showed that both −1031 C and −863 A carriers were independent risk factors to have DU [24]; and one study reported that −1031T/C polymorphism was the risk factor for DU [22]. However, results of our meta-analyses showed no statistically significant association between DU and −1031T/C, −863C/A, −857C/T, and −238G/A polymorphism.

Although no statistical evidence of significant associations were generated from our study, it is likely that TNF-α gene SNPs may increase the susceptibility to DU. The results of our study should be interpreted with caution. Power calculations for the given sample size (overall and subgroups) demonstrated that all the meta-analyses were underpowered. Even with over 1,800 cases and over 3,000 controls, the power was very low. It indicated that the combined sample sizes in our study were still inadequate to detect the association between TNF-α gene SNPs and DU. In addition, gene-gene interactions should be considered. For further study, gene to gene interactions with the generalized multifactor dimensionality reduction method, which has been applied widely [45], would be helpful in understanding the association between TNF-α gene SNPs and DU.

There are some limitations of this meta-analysis. Firstly, the linkage disequilibrium was found among the SNPs analyzed. Haplotype analysis may provide more information in evaluating the association between TNF-α SNPs and DU risk. Secondly, some inevitable bias may exist in the results as our meta-analysis only focused on papers published in English language and studies with full text articles, missing some eligible studies which were unpublished or reported in other languages. Thirdly, there was considerable heterogeneity among the included studies. Heterogeneity may affect the precision of results, despite the use of appropriate meta-analytic techniques with random-effects model.

Nonetheless, to the best of our knowledge, this is the first meta-analysis focusing on the relationship between TNF-α SNPs and DU risk. Our meta-analysis suggests that there is no statistical evidence of significant association between TNF-α SNPs and DU. However, this conclusion should be interpreted with caution as low statistical powers were revealed by power calculations. In future, larger sample-size studies with homogeneous DU patients and well-matched controls are required.

## Supporting Information

Figure S1 Forest plots of all models for −238G/A
(TIF)

Figure S2 Forest plots of all models for −857C/T
(TIF)

Figure S3 Forest plots of all models for −863C/A
(TIF)

Figure S4 Forest plots of all models for −1031T/C
(TIF)

Figure S5 Funnel plots of all models for −308G/A
(TIF)

Figure S6 Funnel plots of all models for −238G/A
(TIF)

Figure S7 Funnel plots of all models for −857C/T
(TIF)

Figure S8 Funnel plots of all models for −863C/A
(TIF)

Figure S9 Funnel plots of all models for −1031T/C
(TIF)

Table S1 PRISMA 2009 Checklist
(DOC)
