# Supplementary figures and images for: Association between TNF α Gene Polymorphisms and the Risk of Duodenal Ulcer: A Meta-Analysis
Source: PLoS One. 2013 Feb 22;8(2):e57167. doi: 10.1371/journal.pone.0057167 (PMC3579801; doi:10.1371/journal.pone.0057167)

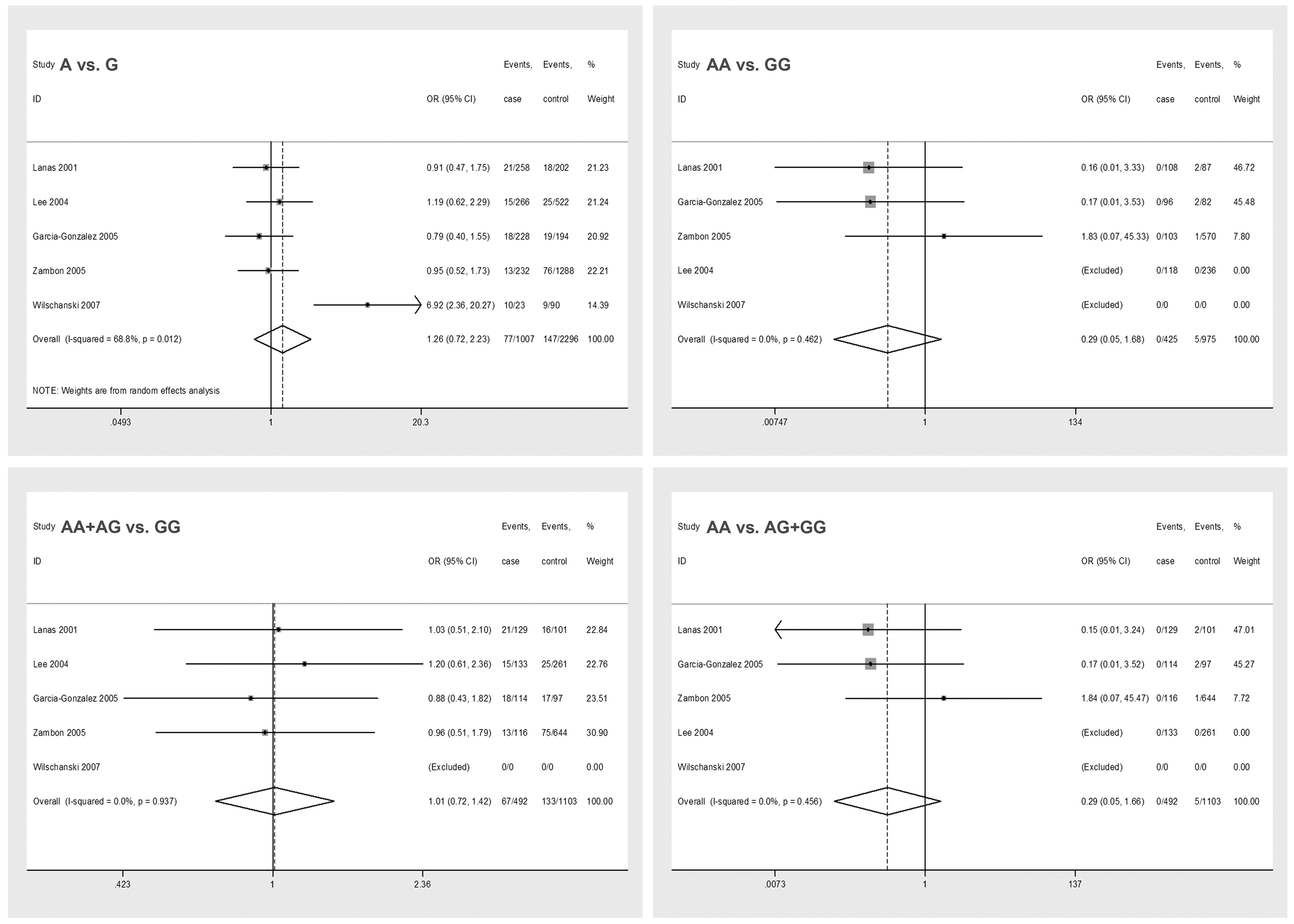

Supplement: Figure S1 — Forest plots of all models for −238G/A (TIF) [file pone.0057167.s001.tif]

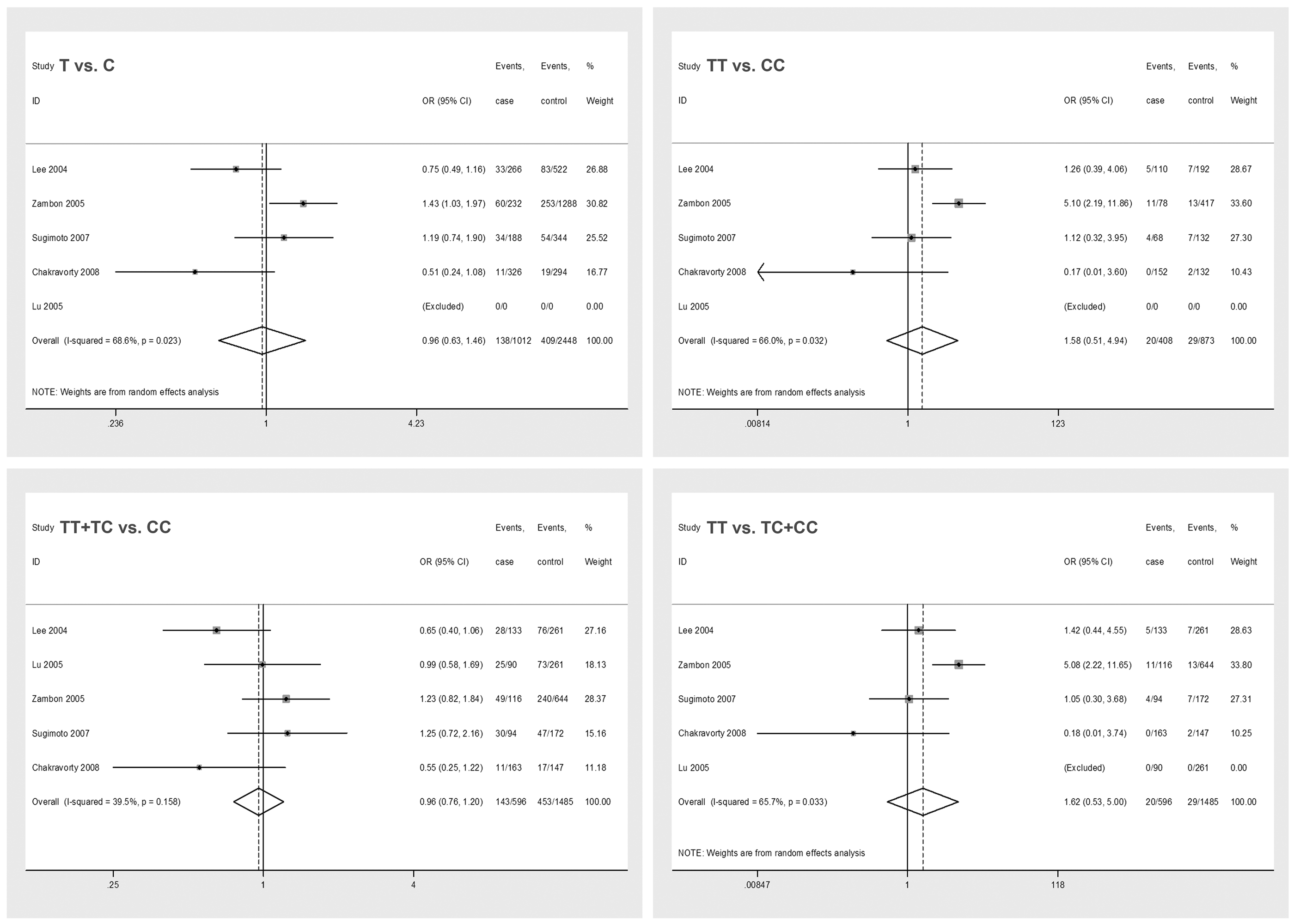

Supplement: Figure S2 — Forest plots of all models for −857C/T (TIF) [file pone.0057167.s002.tif]

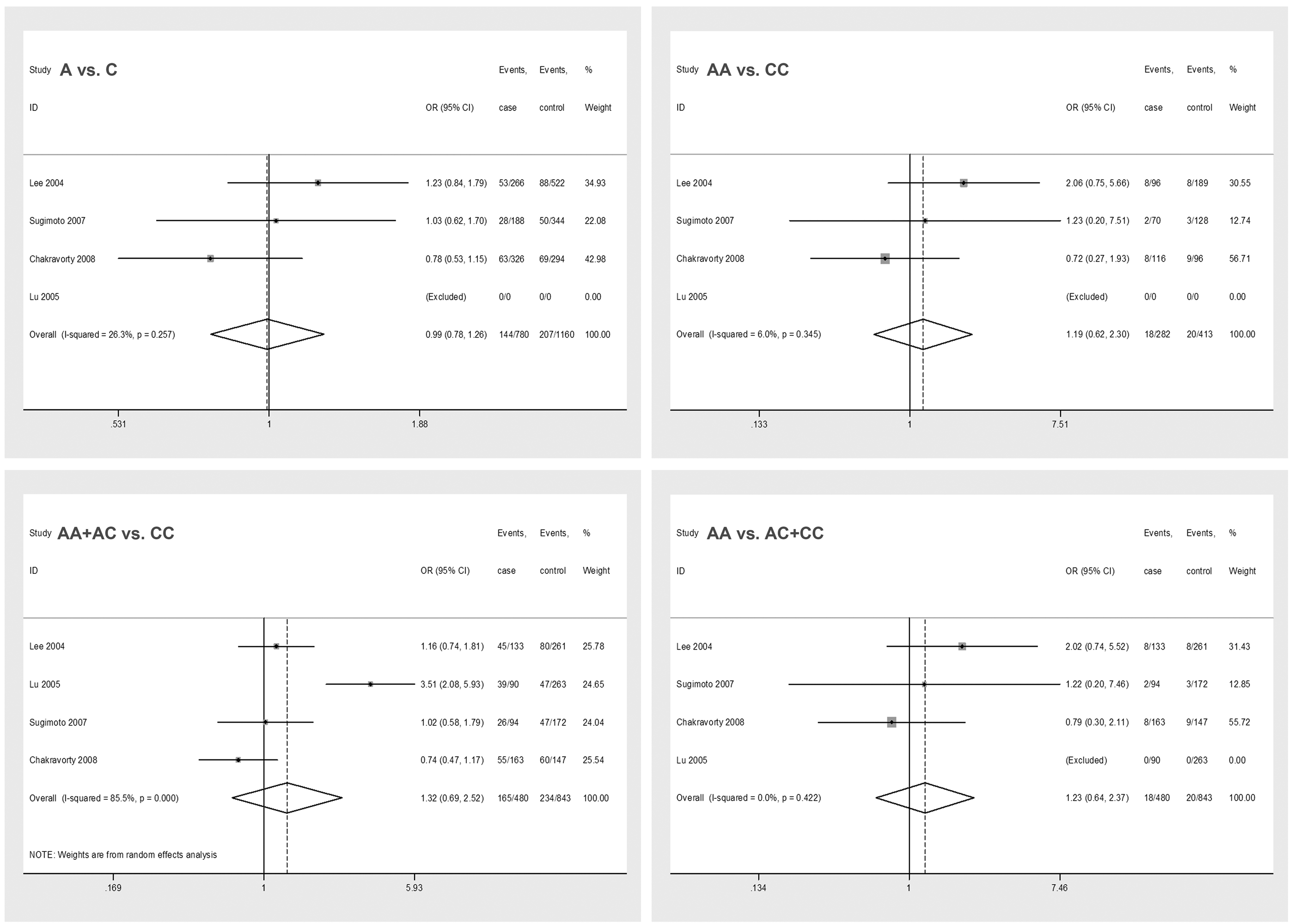

Supplement: Figure S3 — Forest plots of all models for −863C/A (TIF) [file pone.0057167.s003.tif]

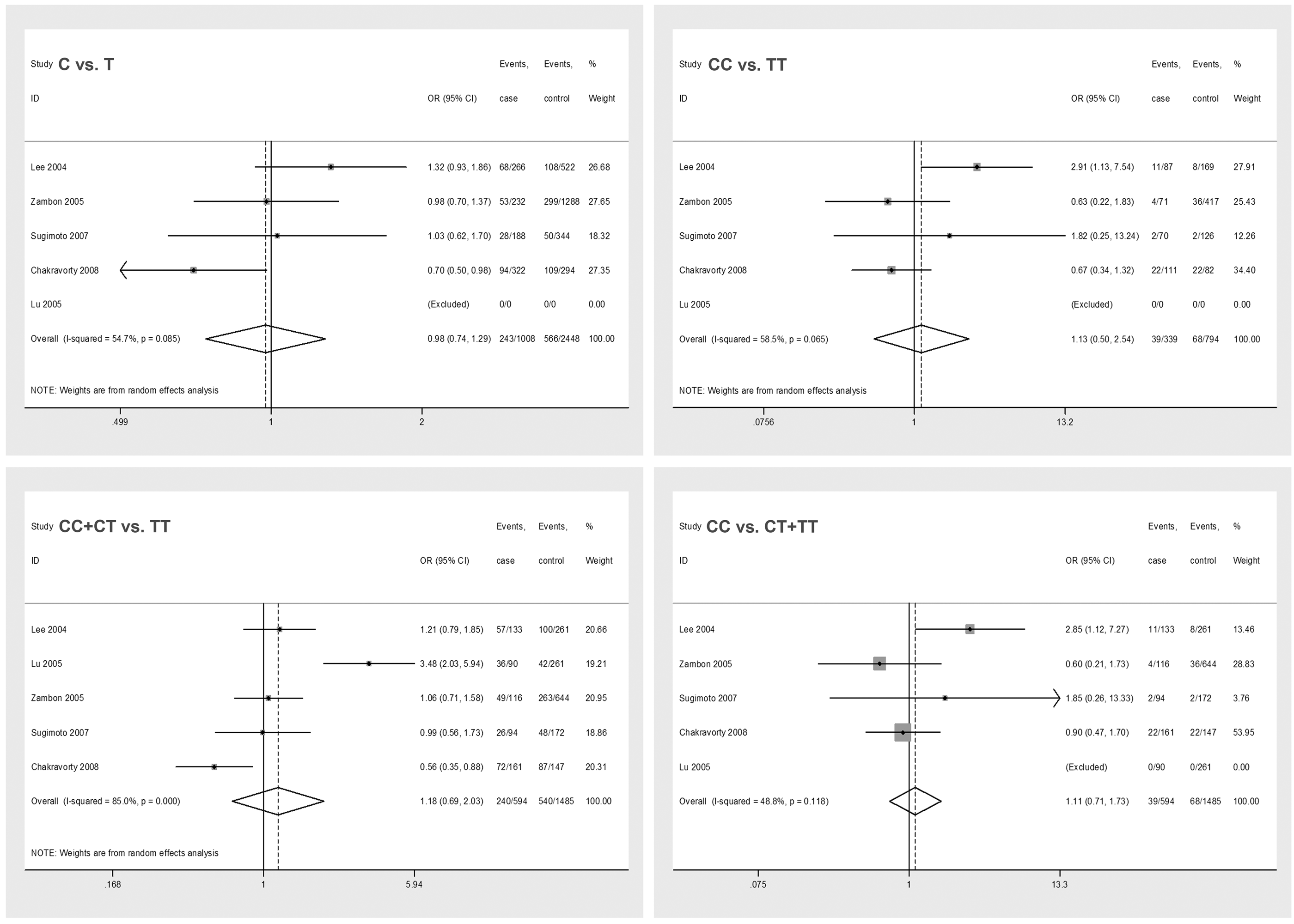

Supplement: Figure S4 — Forest plots of all models for −1031T/C (TIF) [file pone.0057167.s004.tif]

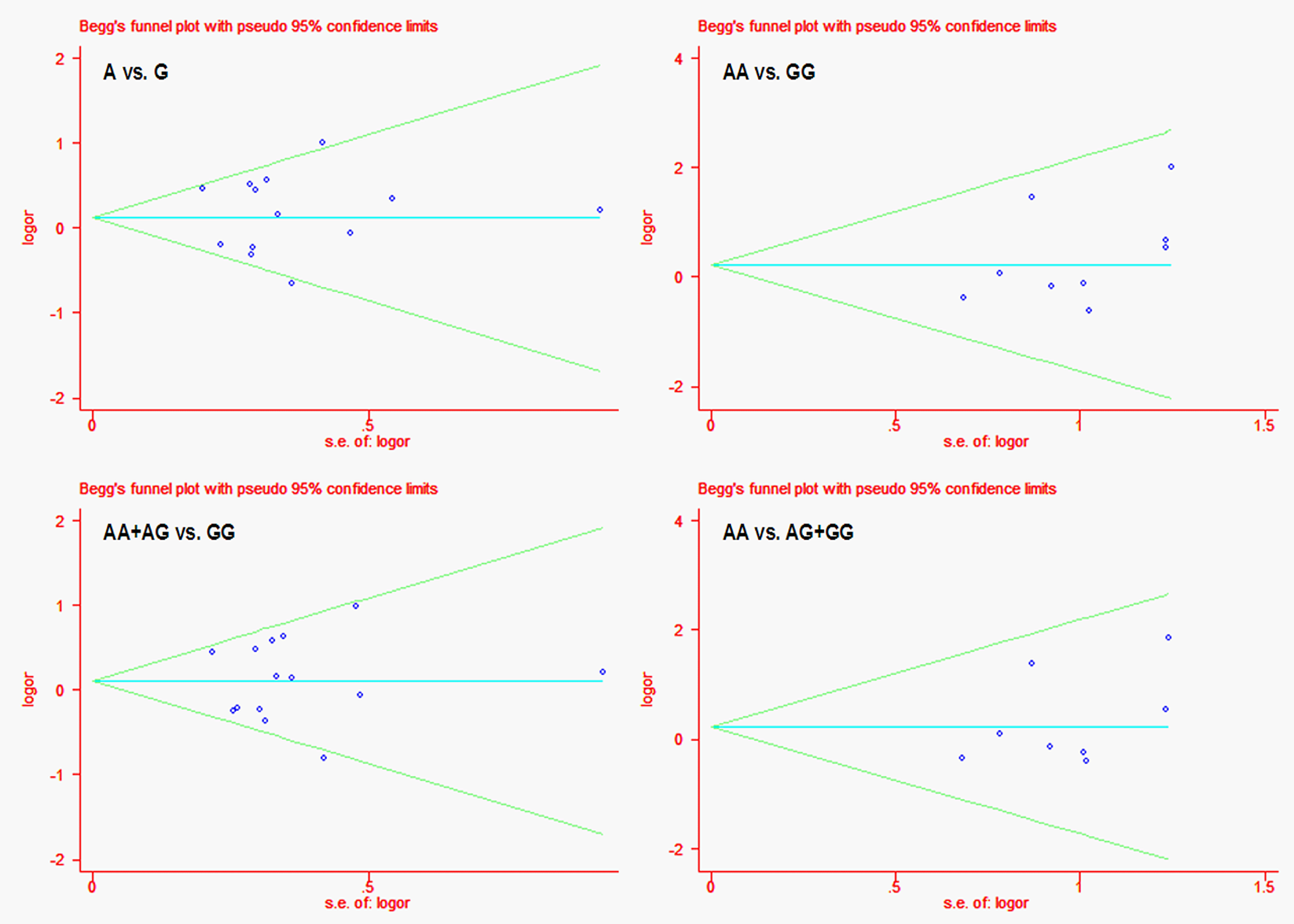

Supplement: Figure S5 — Funnel plots of all models for −308G/A (TIF) [file pone.0057167.s005.tif]

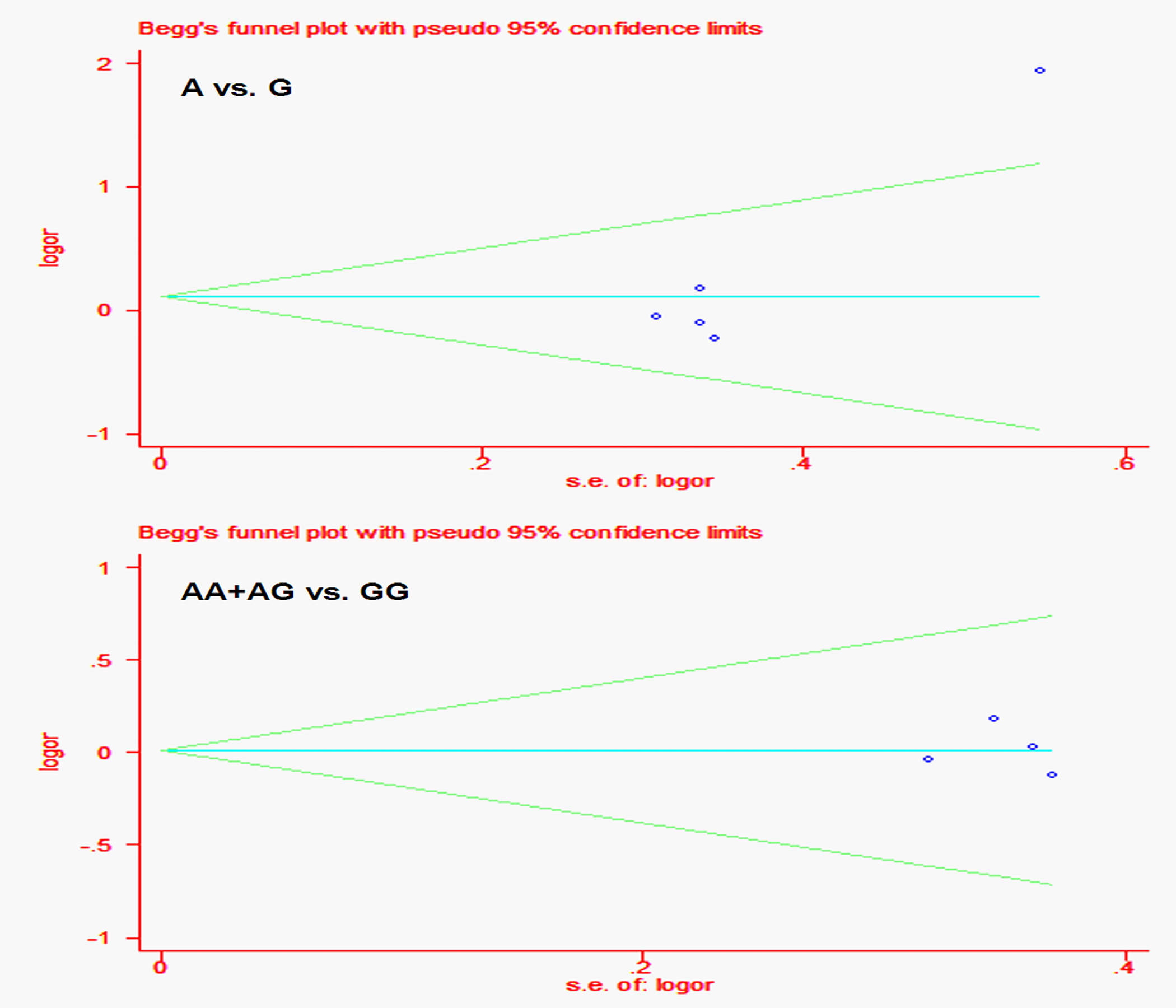

Supplement: Figure S6 — Funnel plots of all models for −238G/A (TIF) [file pone.0057167.s006.tif]

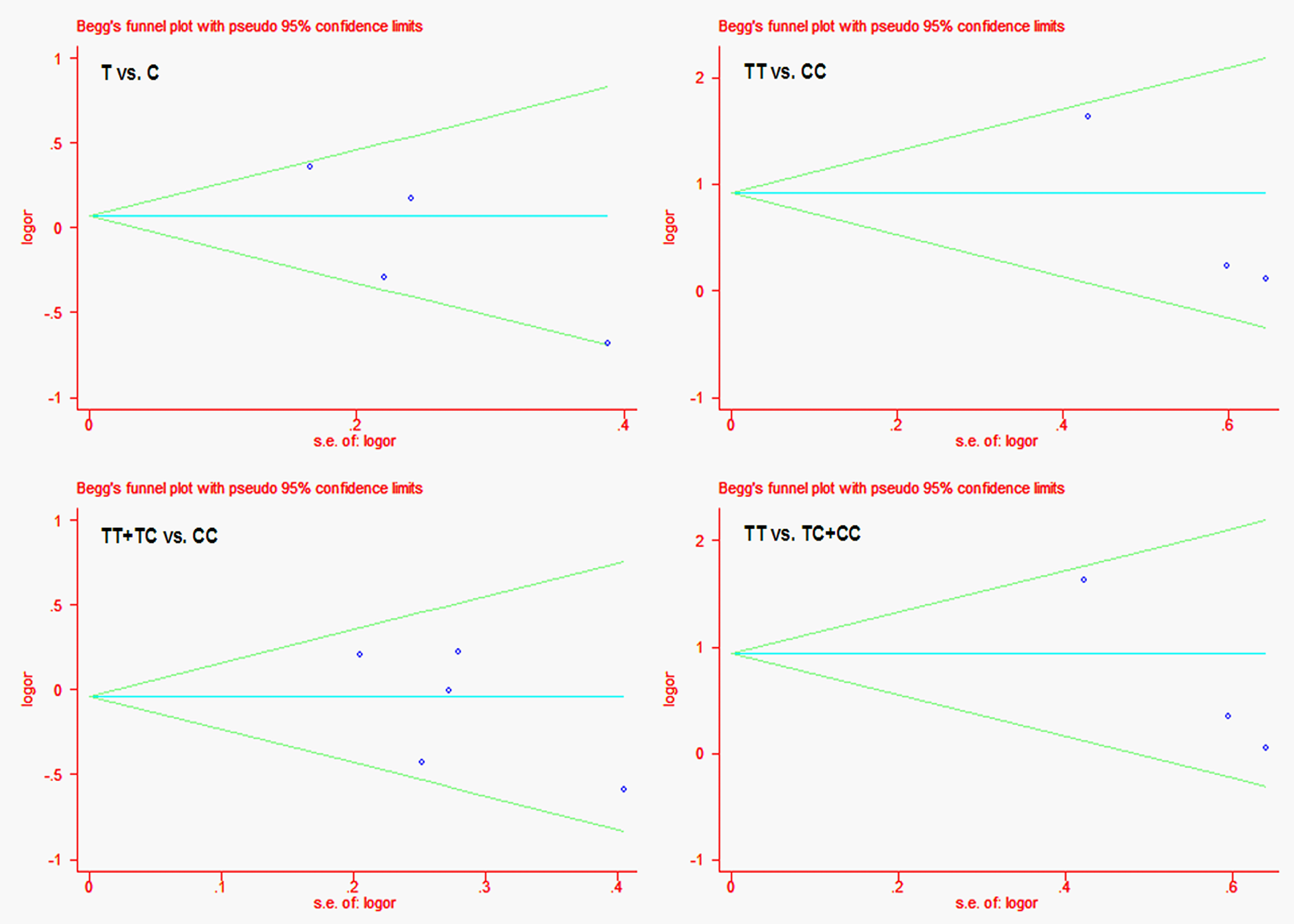

Supplement: Figure S7 — Funnel plots of all models for −857C/T (TIF) [file pone.0057167.s007.tif]

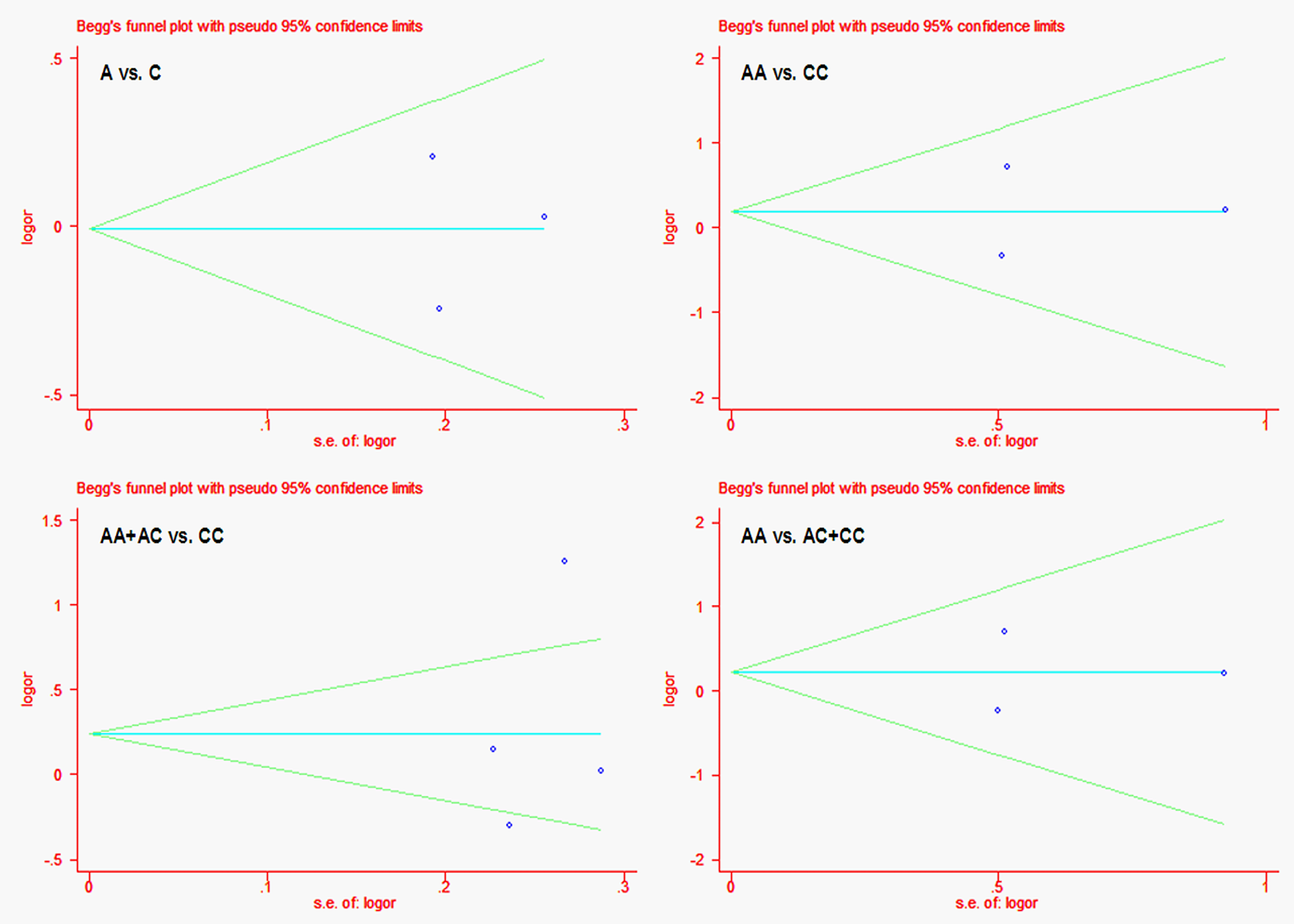

Supplement: Figure S8 — Funnel plots of all models for −863C/A (TIF) [file pone.0057167.s008.tif]

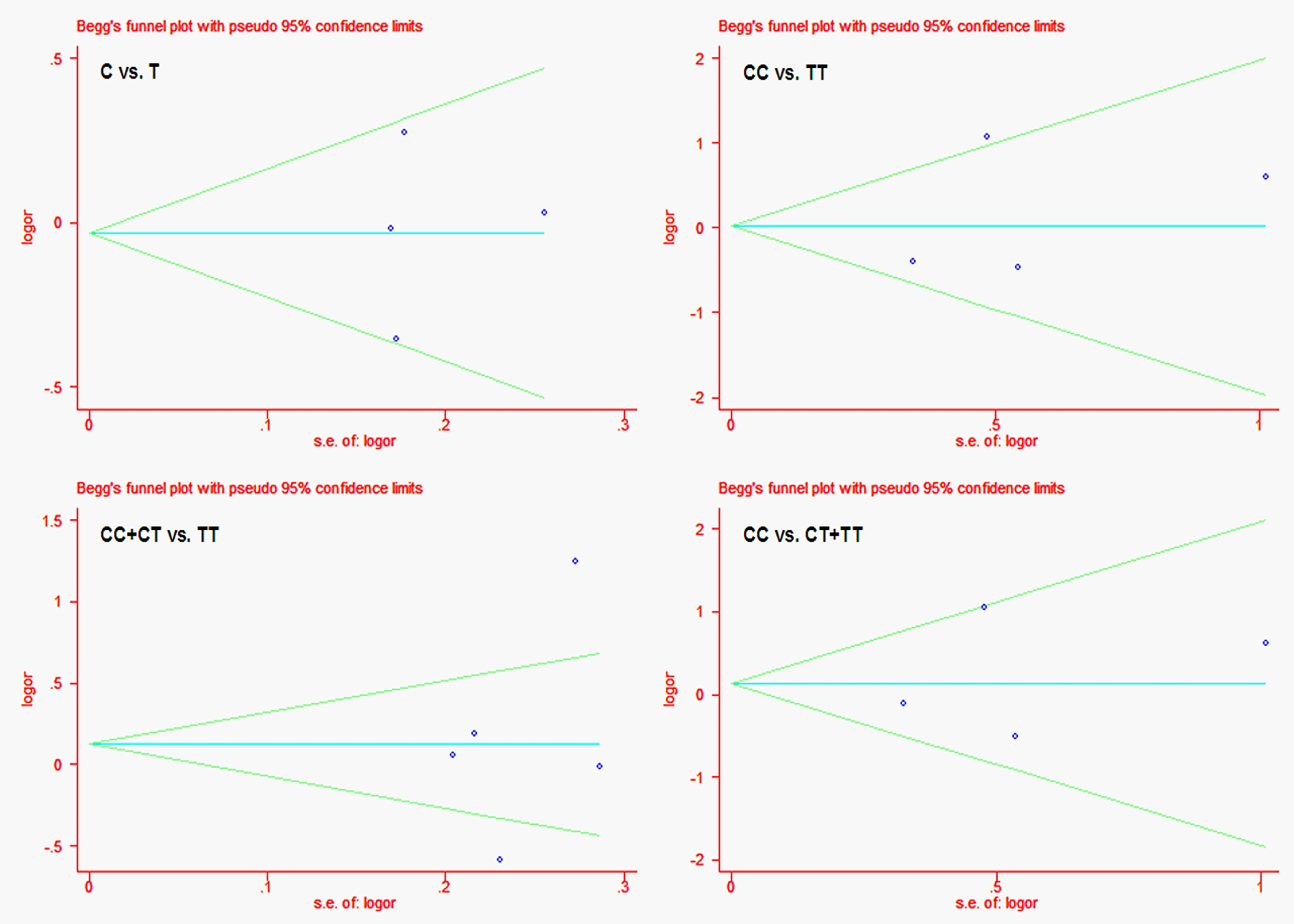

Supplement: Figure S9 — Funnel plots of all models for −1031T/C (TIF) [file pone.0057167.s009.tif]
