# Supplementary material for: Association between TNF α Gene Polymorphisms and the Risk of Duodenal Ulcer: A Meta-Analysis
Source: PLoS One. 2013 Feb 22;8(2):e57167. doi: 10.1371/journal.pone.0057167 (PMC3579801; doi:10.1371/journal.pone.0057167)
Supplement: Table S1 — PRISMA 2009 Checklist (DOC) [file pone.0057167.s010.doc]

| **Section/topic** | **#** | **Checklist item** | **Reported on page #** |
| --- | --- | --- | --- |
| **TITLE** | | |  |
| Title | 1 | Association between TNF α gene polymorphisms and the risk of duodenal ulcer: A meta-analysis | 1 |
| **ABSTRACT** | | |  |
| Structured summary | 2 | Background: Epidemiological studies have evaluated the association between tumor necrosis factor α (TNF-α) single nucleotide polymorphisms (SNPs) and duodenal ulcer (DU), but the results remain inconclusive. The aim of this study was to perform a meta-analysis to investigate a more authentic association between TNF-α SNPs and DU.  Methods: We performed the meta-analysis by searching PubMed, Embase, and Web of Science databases from the first available year to Sep. 5, 2012. Additionally, checking reference lists from identified articles, reviews, and the abstracts presented at related scientific societies meetings were also performed. All case-control studies investigating the association between TNF-α SNPs and DU risk were included. The association was assessed by odds ratio (OR) with 95% confidence interval (CI). Publication bias was analyzed by Begg’s funnel plot and Egger’s regression test.  Results: A total of sixteen studies reporting TNF-α -308G/A, -1031T/C, -863C/A, -857C/T, and -238G/A polymorphism were included in our final meta-analysis. There was no statistically significant association between -308G/A polymorphism and DU in the overall study population, as well as subgroup analyses by ethnicity, study design, and H. pylori status. As for -1031T/C, -863C/A, -857C/T, and -238G/A, results of our meta-analyses showed no statistical evidence of significant association. Power calculation on the combined sample size showed that the statistical powers were all lower than 80% for all the meta-analyses.  Conclusions: The data suggests that there is no statistical evidence of significant association between the studied TNF-α SNPs and DU. However, this conclusion should be interpreted with caution as low statistical powers were revealed by power calculations. In future, larger sample-size studies with homogeneous DU patients and well-matched controls are required. | 2 |
| **INTRODUCTION** | | |  |
| Rationale | 3 | Tumor necrosis factor α (TNF-α) has been reported to have the proinflammatory activity and the capability of inhibiting gastric acid secretion. TNF-α, which is produced by macrophages, monocytes, neutrophils, T-cells and NK-cells after stimulation, is a pro-inflammatory cytokine and plays a role in cell immunity. The TNF-α gene is located in the class III region of the major histocompatibility complex (MHC) on chromosome 6. It is suggested that polymorphisms in the regulatory region could influence the expression of TNF-α, and thereby increased the susceptibility of H. pylori infection. In 1999, Kunstmann et al. firstly reported the association of specific genotype of the TNF-α gene with the susceptibility to DU. Thereafter, a variety of epidemiological studies have evaluated the association between DU and TNF-α promoter polymorphisms, including -308G/A, -1031T/C, -863C/A, -857C/T, -238G/A, -376 G/A and -806 C/T. However, results of different studies have been inconsistent. In addition, the sample size in each of published studies was relatively small, which limited the credibility of results. | 3 |
| Objectives | 4 | The present meta-analysis was designed to derive a more precise estimation of the association between TNF-α single nucleotide polymorphisms (SNPs) and DU. | 3-4 |
| **METHODS** | | |  |
| Protocol and registration | 5 | No protocol and registration. |  |
| Eligibility criteria | 6 | (1) studies on the relationship between TNF-α gene polymorphisms and DU, including -308G/A, -1031T/C, -863C/A, -857C/T, -238G/A, -376 G/A and -806 C/T; (2) published case-control studies; (3) studies with full text articles; (4) sufficient data for estimating an odds ratio (OR) with 95% confidence interval (CI). | 4 |
| Information sources | 7 | PubMed, Embase and Web of Science were searched from the first available year to Sep. 5, 2012, as well as hand searching of the references of identiﬁed articles were performed. | 4 |
| Search | 8 | Search strategy: (“tumor necrosis factor α” OR “TNF-α”) AND (“polymorphism” OR “mutation” OR “variant” OR “genotype”) AND (“duodenal ulcer” OR “peptic ulcer”). | 4 |
| Study selection | 9 | Two investigators (Zhang BB and Yin YW) screened each of the titles, abstracts, and full texts to determine inclusion independently. The results were compared and disagreements were resolved by consensus. | 4 |
| Data collection process | 10 | Information was carefully extracted from all included publications independently by two of the authors (Zhang BB and Yin YW) according to the inclusion criteria listed above. Disagreement was resolved by consensus. If these two authors could not reach a consensus, another author (Sun QQ) was consulted. | 4 |
| Data items | 11 | The following data were collected from each study: first author’s name, publication date, country, ethnicity, study design (source of controls), and evidence of Hardy-Weinberg equilibrium (HWE) (P<0.05 of HWE was considered significant), respectively. Different ethnicities were categorized as Caucasian, Asian, African, American Indian, and mixed. Study design was stratiﬁed to population-based (PB) studies and hospital-based (HB) studies. Total numbers of cases and controls, and frequency of -308G/A, -1031T/C, -863C/A, -857C/T, -238G/A, -376 G/A and -806 C/T mutation in cases and controls regardless of H. pylori status were extracted. When studies reported genotype distributions for H. pylori negative and H. pylori positive only, we also extracted data of each group separately for subgroup analyses. | 4-5 |
| Risk of bias in individual studies | 12 | The quality of included studies was evaluated independently by two authors (Zhang BB and Yin YW) of this article according to the Newcastle-Ottawa Scale (NOS). | 5 |
| Summary measures | 13 | The principal summary measures are odds ratios (ORs) and 95% confidence intervals (CIs). | 5 |
| Synthesis of results | 14 | Combined ORs with their 95% CIs were calculated respectively for four genetic models: allelic model (2 allele vs. 1 allele), additive model (2/2 vs. 1/1), dominant model (1/2+2/2 vs. 1/1), and recessive model (2/2 vs. 1/2+1/1), in which 2 indicates the minor allele. Between-study heterogeneity was assessed by the Q-test and I^2^ statistic, P<0.10 and I^2^>50% indicated evidence of heterogeneity. The ORs were pooled through a fixed effects model, using the Mantel-Haenszel approach when no heterogeneity was observed among studies. Otherwise, a random effects model was adopted. | 5 |

Page 1 of 2

| **Section/topic** | **#** | **Checklist item** | **Reported on page #** |
| --- | --- | --- | --- |
| Risk of bias across studies | 15 | An estimate of potential publication bias was carried out by Begg’s funnel plot and Egger’s regression test (p < 0.05 was considered representative of statistically signiﬁcant publication bias) | 5-6 |
| Additional analyses | 16 | Subgroup analyses were performed by ethnicity, study design, and H. pylori status. Power analysis was performed using Quanto software package. | 5-6 |
| **RESULTS** | | |  |
| Study selection | 17 | Two hundred and forty-two potentially relevant articles studies were identified after the searching. Based on our inclusion criteria, a total of nineteen studies were included in qualitative synthesis. Three studies were excluded for further meta-analysis for providing no origin data to calculating ORs and 95%CIs. Hence, sixteen articles were included in the meta-analysis | 6 |
| Study characteristics | 18 | Table 1 shows the studies included in the meta-analysis and their main characteristics. | 14 |
| Risk of bias within studies | 19 | The NOS results were shown in Table 1. | 14 |
| Results of individual studies | 20 | The main results of individual studies were shown in Figure 2, S1-S4, respectively. |  |
| Synthesis of results | 21 | The combined effects were as follows: for A allele vs. G allele: OR=1.11, 95%CI=0.87~1.42; for AA vs. GG: OR=1.19, 95%CI=0.69~2.05; for AA+AG vs. GG: OR=1.10, 95%CI=0.85~1.41; and for AA vs. AG+GG: OR=1.22, 95%CI=0.70~2.11 (Fig. 2). When meta-analysis was performed to assess association between -308G/A polymorphism and DU based on H. pylori status, no statistical evidence of significant association was found in all genetic models for both H. pylori positive subgroup and H. pylori negative subgroup. When stratiﬁed by ethnicity and study design, no statistically significant associations were found (Table 2). As for the association of TNF-α -238G/A, -1031T/C, -863C/A, -857C/T and DU susceptibility, there were no statistical evidence of significant association between all the above SNPs and DU susceptibility (Fig. S1-S4). Power calculation on the pooled frequencies showed that the statistical powers were all lower than 80% for all the above meta-analyses | 6-7 |
| Risk of bias across studies | 22 | The shapes of the funnel plots did not reveal any evidence of obvious asymmetry visually (Fig. S5-S9). However, statistical evidence of publication bias were found using Egger’s regression test for allelic model of -238G/A mutation and recessive model of -857C/T mutation. The results were as follows: for -308G/A mutation (P=0.83 for allelic model, P=0.22 for additive model, P=0.98 for dominant model, and P=0.23 for recessive model, respectively); for -238G/A mutation (P=0.02 for allelic model, P=0.89 for dominant model, respectively); for -1031T/C mutation (P=0.84 for allelic model, P=0.61 for additive model, P=0.52 for dominant model, and P=0.72 for recessive model, respectively); for -863C/A mutation (P=0.98 for allelic model, P=0.97 for additive model, P=0.62 for dominant model, and P=0.99 for recessive model, respectively); for -857C/T mutation (P=0.18 for allelic model, P=0.07 for additive model, P=0.29 for dominant model, and P=0.008 for recessive model, respectively). | 7 |
| Additional analysis | 23 | **Subgroup analysis:** When meta-analysis was performed to assess association between -308G/A polymorphism and DU based on H. pylori status, no statistical evidence of significant association was found in all genetic models for both H. pylori positive subgroup and H. pylori negative subgroup. When stratiﬁed by ethnicity and study design, no statistically significant associations were found. | 6-7 |
| **DISCUSSION** | | |  |
| Summary of evidence | 24 | A total of sixteen publications were included in our meta-analysis. Our meta-analyses did not show evidence for association between the TNF-α SNPs and DU. | 8 |
| Limitations | 25 | **Limitations:** Firstly, the linkage disequilibrium was found among the SNPs analyzed. Haplotype analysis may provide more information in evaluating the association between TNF-α SNPs and DU risk. Secondly, some inevitable bias may exist in the results as our meta-analysis only focused on papers published in English language and studies with full text articles, missing some eligible studies which were unpublished or reported in other languages. Thirdly, there was considerable heterogeneity among the included studies. Heterogeneity may affect the precision of results, despite the use of appropriate meta-analytic techniques with random-effects model. | 9 |
| Conclusions | 26 | **Conclusion：**There was no evidence of significant association between TNF-α SNPs and DU. However, this conclusion should be interpreted with caution as low statistical powers were revealed by power calculations. In future, larger sample-size studies with homogeneous DU patients and well-matched controls are required. | 9 |
| **FUNDING** | | |  |
| Funding | 27 | No current external funding sources for this study. | 10 |

*From:*  Moher D, Liberati A, Tetzlaff J, Altman DG, The PRISMA Group (2009). Preferred Reporting Items for Systematic Reviews and Meta-Analyses: The PRISMA Statement. PLoS Med 6(6): e1000097. doi:10.1371/journal.pmed1000097

For more information, visit: **www.prisma-statement.org**.

Page 2 of 2
